# UAV-Borne Dual-Band Sensor Method for Monitoring Physiological Crop Status

**DOI:** 10.3390/s19040816

**Published:** 2019-02-17

**Authors:** Lili Yao, Qing Wang, Jinbo Yang, Yu Zhang, Yan Zhu, Weixing Cao, Jun Ni

**Affiliations:** National Engineering and Technology Center for Information Agriculture, Key Laboratory for Crop System Analysis and Decision Making, Ministry of Agriculture, Jiangsu Key Laboratory for Information Agriculture, Nanjing Agricultural University, Nanjing 210095, Jiangsu, China; 2017201083@njau.edu.cn (L.Y.); 2016101037@njau.edu.cn (Q.W.); 2018101166@njau.edu.cn (J.Y.); zhangyu@njau.edu.cn (Y.Z.); yanzhu@njau.edu.cn (Y.Z.)

**Keywords:** CFD, airflow field test, monitoring method, spectral sensor, crop growth

## Abstract

Unmanned aerial vehicles (UAVs) equipped with dual-band crop-growth sensors can achieve high-throughput acquisition of crop-growth information. However, the downwash airflow field of the UAV disturbs the crop canopy during sensor measurements. To resolve this issue, we used computational fluid dynamics (CFD), numerical simulation, and three-dimensional airflow field testers to study the UAV-borne multispectral-sensor method for monitoring crop growth. The results show that when the flying height of the UAV is 1 m from the crop canopy, the generated airflow field on the surface of the crop canopy is elliptical, with a long semiaxis length of about 0.45 m and a short semiaxis of about 0.4 m. The flow-field distribution results, combined with the sensor’s field of view, indicated that the support length of the UAV-borne multispectral sensor should be 0.6 m. Wheat test results showed that the ratio vegetation index (RVI) output of the UAV-borne spectral sensor had a linear fit coefficient of determination (R^2^) of 0.81, and a root mean square error (RMSE) of 0.38 compared with the ASD Fieldspec2 spectrometer. Our method improves the accuracy and stability of measurement results of the UAV-borne dual-band crop-growth sensor. Rice test results showed that the RVI value measured by the UAV-borne multispectral sensor had good linearity with leaf nitrogen accumulation (LNA), leaf area index (LAI), and leaf dry weight (LDW); R^2^ was 0.62, 0.76, and 0.60, and RMSE was 2.28, 1.03, and 10.73, respectively. Our monitoring method could be well-applied to UAV-borne dual-band crop growth sensors.

## 1. Introduction

Accurate management of crop water and fertilizer in crop fields is an important prerequisite for ensuring high yield and quality of crops, sustainable use of cultivated land, and healthy development of the environment [1]. High-throughput, accurate, and real-time acquisition of crop-growth information is an important basis for the accurate management of crop water and fertilizer [2]. Monitoring technology based on the characteristics of the reflection spectrum has the advantages of being nondestructive, providing real-time information, and delivering high-efficiency analysis. Thus, it is widely used in crop-growth parameter acquisition. Various research institutions have developed spectral sensors to monitor crop growth, providing effective technical support for field-crop production management [3,4,5].

In 2004, Moya et al. [6] designed a chlorophyll-fluorescence test device, which uses sunlight as a light source. During the test, the leaf blade was required to be in a relatively static state, and the reflection=spectrum information of chlorophyll fluorescence in the 510 and 570 nm bands could be obtained from a short distance. Quantitative inversion of chlorophyll fluorescence could be achieved by the physiological reflectance index (PRI) calculated from the test results. In 2010, Ryu et al. [7] developed a normalized vegetation index spectroscopy sensor to achieve the inversion of vegetation-growth indicators. It has its own LED light source for illumination. When the test height was less than 3 m, the best test results could be achieved. Several commercial instruments are currently available for crop-growth monitoring. For example, the Greenseeker spectral sensor designed by Trimble USA can obtain the spectral information of reflection characteristics in crop canopy red and near-infrared bands and calculate the relevant vegetation index. For this device, the test distance should be kept at a height of 60–180 cm from the canopy [8,9]. The ASD FieldSpec4 spectrometer developed by American ASD Company can realize reflection-spectrum acquisition of the 350–2500 nm crop-canopy band, the data information is rich, and accuracy is high. Relying on sunlight as a light source, the test needs to be carried out at noon without wind or clouds, the test height should be kept between 30–120 cm, and the crop canopy needs to remain relatively static [10,11,12]. Holland Scientific designed and developed active light-source spectral sensors for monitoring crop growth, such as Crop Circle and Rapidscan. These instruments can emit light and receive reflection-spectrum information of a crop canopy in real time through their own light-source system. The test height should be kept within 3 m from the canopy, and the canopy structure needs to maintain a steady state [13,14,15,16,17]. These spectral crop-growth monitoring devices are simple in operation, easy to carry, high in test accuracy, intuitive in results, and can provide nondestructive access to crop-growth information, but they also have shortcomings, such as a small monitoring range, high labor intensity, and discontinuous monitoring, which cannot provide high-throughput information and real-time decision-making for large-scale crop-production management in the field.

Unmanned-aerial-vehicle (UAV) operation is highly efficient, flexible, easy, and has strong terrain applicability. Thus, UAVs are widely used in agricultural information-acquisition platforms [12], but so far there are few studies on the use of UAV-borne spectral sensors for monitoring crop growth. Krienke et al. attempted to measure the normalized vegetation index of lawn using a MikroKopterOktoKopter XL UAV equipped with a RapidScan CS-45 spectroscopy sensor. Flight height was maintained at 0.5–1.5 m above the lawn. However, test results were poor because the disturbance of the turf canopy from the downwash airflow field was ignored [18]. Shafian et al. mounted an image sensor on a fixed-wing UAV and collected the image information of a sorghum planting area at an altitude of 120 m. Pix4D software was used to splice, correct, and extract vegetation indices from each acquired image. The leaf area index (LAI) value of sorghum was simultaneously sampled and tested. The results show that the Normalized Difference Vegetation Index (NDVI) value extracted from the acquired image information had a higher linear fit with the sorghum LAI value obtained with the sampling test [19]. Schirrmann et al. used a UAV to work at an altitude of 50 m and obtain an RGB image of the wheat growth period. The acquired image was calibrated by Agisoft lens software, the distortion correction was modeled by Brown distortion model, and the final image mosaic and surface model generation results were improved by radiation pretreatment, from which the information such as crop coverage and plant height were extracted [20]. Zheng et al. used an OktoXL UAV equipped with a Cuubert UHD 185 hyperspectral camera to acquire hyperspectral images of a rice canopy at an altitude of 50 m. Images in the acquisition results were corrected using ENVI software. By comparing the synchronous test results of a ground-object spectrometer and the agronomic parameters obtained from laboratory chemical analysis, they proved that the image information acquired by the UAV platform equipped with imaging instruments could be used for the quantitative inversion of agronomic crop parameters [21]. Stroppianaet al. acquired a large number of multispectral images at a height of 70 m from the ground by using a 3DRobotics SOLO quadrotor UAV equipped with a Parrot Sequoia multispectral camera. By screening the acquired images, and then correcting and extracting the vegetation index, they proposed an automatic classification method for weeds and crops, which can be used for the classification and management of specific weeds in the field [22]. However, these studies simply installed image sensors on UAVs to obtain crop images from a high altitude. Although the influence of the UAV downwash airflow field is small, captured images can only be stored in the memory. Scientific-research personnel are required to use special software for image correction, cropping, splicing, enhancement, and other offline processing, and then analyze the relationship between the images and crop-growth parameters. The process is complex, requires specialized knowledge, and cannot acquire information in real time, which is not conducive to popularization [23,24].

In this paper, we studied the dual-band crop-growth sensor independently developed by Nanjing Agricultural University [25]. First, we investigated the monitoring method of the UAV-borne dual-band crop-growth sensor based on its spectral-monitoring mechanism and structural-design features. Then, we analyzed the spatial-distribution characteristics of the airflow field under the low-altitude hovering operation of the UAV. Finally, we built a UAV-borne crop-growth monitoring system to achieve high throughput and real-time access to rice- and wheat-growth information.

## 2. Materials and Methods

### 2.1. Test Equipment

#### 2.1.1. Dual-Band Crop-Growth Sensor

**Figure 1:**
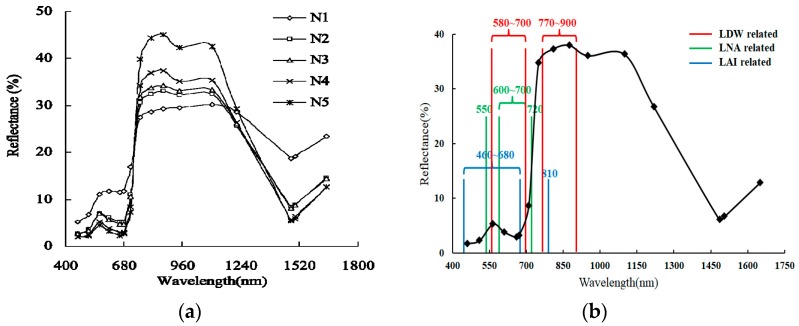
Characteristic curve of the spectral reflectivity of wheat canopy. (**a**) Multispectral reflectivity of wheat canopy under application of different amounts of nitrogen, and (**b**) characteristic spectral bands of agronomic wheat parameters.

There is a certain relationship between crop growth and the spectral reflectance of the crop canopy. As shown in Figure 1, the reflectivity of a wheat canopy is relatively low when the band is under 710 nm. Reflectivity linearly rises in the 710–760 nm band, and reaches a comparatively high level in the 760–1210 nm band. Wheat-canopy reflectivity shows significant differences between different nitrogen levels for the 460–730 and 760–1210 nm bands. In the 460–730 nm band, spectral reflectivity is negatively correlated with the amount of nitrogen fertilizer, with reflectivity being the lowest at N5 and highest at N1. In the 760–1210 nm band, spectral reflectivity is positively correlated with the amount of nitrogen fertilizer, with reflectivity being the highest at N5 and lowest at N1. The 710–760 nm band is an apparent transition zone. The spectral reflectivity of the wheat canopy at above 1150 nm is not very susceptible to the amount of nitrogen fertilizer, and there is little difference between spectral reflectivity at N2–N5. According to currently available research, there is a good linear relationship between leaf nitrogen accumulation (LNA) and spectral-reflectivity changes near 550, and 600–700 and 720 nm; there is a good linear relationship between leaf dry weight (LDW), and spectral-reflectivity changes at 580–700 and 770–900 nm; the spectral-reflectivity change at 460–680 nm and near 810 nm is closely correlated the LAI. Considering the sensitive bands of the three agronomic parameters, crop growth can be well-inverted with the ratio vegetation index (RVI) index constructed using the 730 and 810 nm bands [26,27,28].

**Figure 2:**
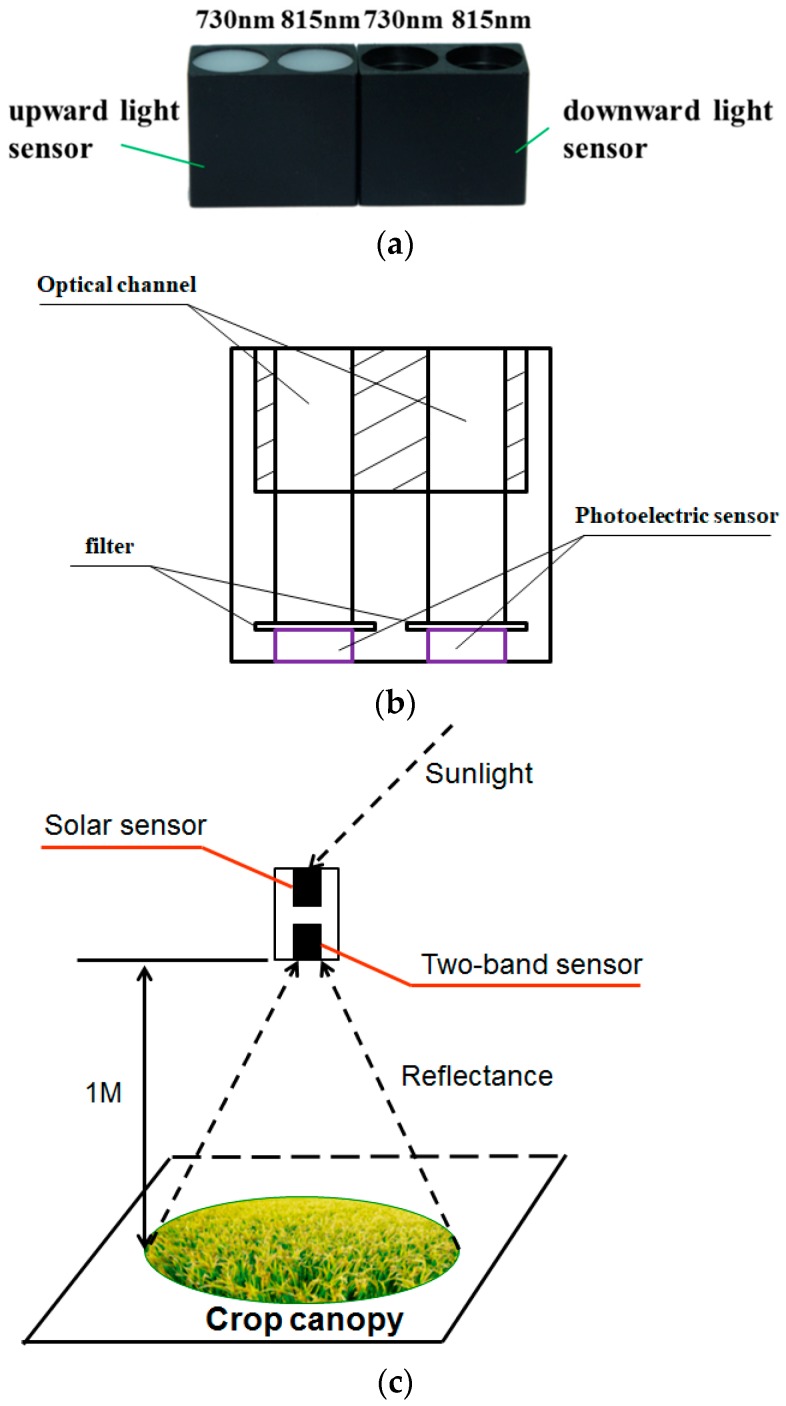
Introduction of dual-band crop-growth sensor. (**a**) Sensor; (**b**) sensor structure; (**c**) schematic diagram of dual-band crop-growth sensor test.

The dual-band crop-growth sensor was designed by Nanjing Agricultural University, it is equipped with a dual-band detection lens, and its structure can be divided into an upward light sensor and a downward light sensor, as shown in Figure 2. The upward light sensor is used to acquire sunlight-radiation information at 730 and 815 nm wavelengths, and the downward light sensor was configured to receive crop-canopy-reflected light-radiation information of a corresponding wavelength. The structure is shown in Figure 2. It is packaged in a nylon case and weighs 11.34 g, with a test field of view of 27°. The crop-canopy RVI can be output in real time, and wireless transmission can achieve long-distance transmission and analysis.

With sunlight as the light source, dual-band crop-growth sensors require the testing object (i.e., wheat canopy) to remain relatively static so that the canopy presents the Lambertian reflection characteristics and the field of view of the sensor points vertically downward. During measurement, optical-radiation energy is converted to electric-energy signals by the sensor. Therefore, to ensure high sensitivity, measuring height should be maintained at 1.0–1.5 m above the canopy. The principle is shown in Figure 2.

#### 2.1.2. ASD FieldSpec HandHeld 2 Handheld Spectrometer

Developed by American Analytical Spectral Devices (AS, made by Advanced Systems Development, Inc., Alexandria, VA, America), the ASD FieldSpec HandHeld 2 handheld spectrometer can be used for the reflection-spectrum acquisition of different objects such as crops, marine organisms, and minerals. The device has the advantages of portability, simple operation, and accurate results. Test wavelength range is 325–1075 nm, wavelength accuracy is ±1 nm, spectral resolution is less than 3 nm, and test field of view is 25° [29,30].

#### 2.1.3. LAI-2200C Vegetation Canopy Analyzer

The LAI-2200C (Made by LI-COR, Lincoln, NE, USA) is a vegetation-canopy analyzer manufactured by LI-COR, United States. The analyzer is light in weight, consumes little power, and is very suitable for outdoor measurements. In addition, the analyzer can work independently, perform unattended long-term continuous measurement, and automatically record data. The measurement principle is to measure the transmitted light at five angles above and below the vegetation canopy by using the “fisheye” optical sensor (which has a 148° vertical field of view and a 360° horizontal field of view), and calculate canopy-structure parameters such as LAI, average leaf dip angle, void ratio, and aggregation index by using the radiation-transfer model of a vegetation canopy [31,32,33]. 

#### 2.1.4. Three-Dimensional Airflow Field Tester

**Figure 3:**
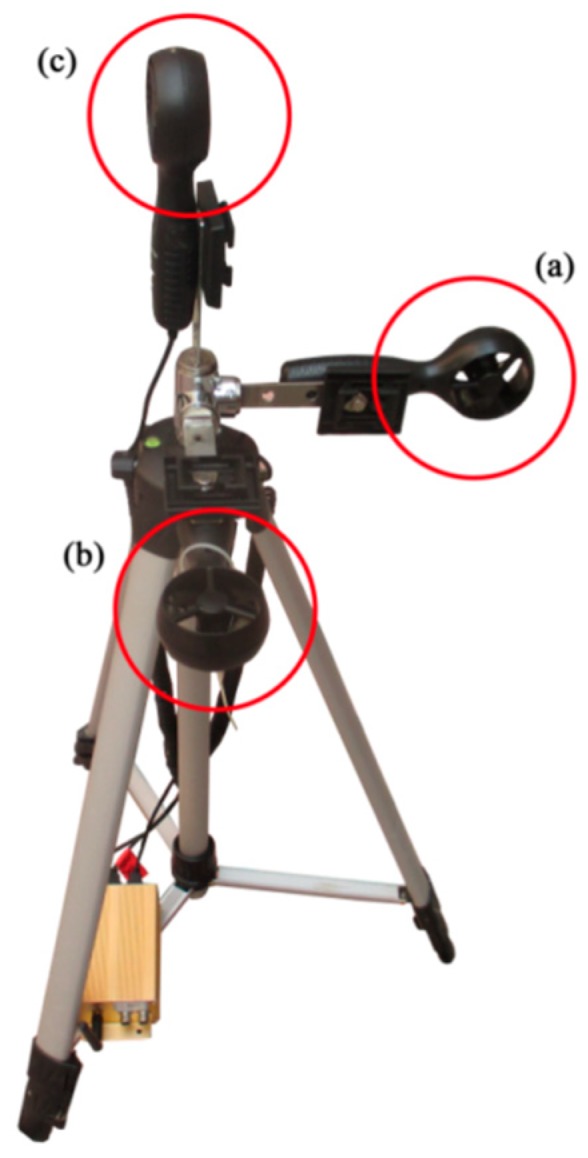
Structure of three-dimensional airflow field tester. (**a**) X-axial velocity test axis; (**b**) Y-axial velocity test axis; (**c**) Z-axial velocity test axis.

The three-dimensional (3D) airflow field tester (South China Agricultural University, Guangzhou, China) uses three wind-speed sensors to test X-, Y-, and Z-axial airflow velocity. The test results are transmitted to a computer through the Zigbee module in real time. The power consumption of the sensor is little, and it can maintain continuous operation for a long time. The center point of the three test axes was fixed to a height of 60 cm from the ground, and the distance from each wind speed sensor to the center point was 15 cm. The three test axes were kept perpendicular to each other. The structure is shown in Figure 3.

### 2.2. Research Methods

When the dual-band crop-growth sensor is measuring, test height is required to maintain a distance of 1–1.5 m from the crop canopy, and the crop canopy must remain relatively static, exhibiting Lambertian reflection characteristics. However, while the UAV is hovering at a low altitude, rotors rotate at a high speed, which causes the surrounding airflow to shrink and contract, forming an airflow field; this airflow field acts on the crop canopy, causing disturbance to it, destroying the Lambertian reflection characteristics of the crop canopy, affecting the test results, and even preventing completion of the test. Therefore, when a UAV is equipped with a dual-band crop-growth sensor for crop-growth monitoring, it is necessary to consider the disturbance effect of the downwash airflow field on the crop canopy.

To solve this problem, we analyzed the spatial distribution of the UAV rotor downwash airflow field by using computational fluid dynamics (CFD) and 3D airflow field testers. We then determined an acceptable deployment location for the dual-band crop-growth sensor based on the surface velocity and distribution range of the airflow field.

#### 2.2.1. Airflow-Field Numerical Simulation

**Figure 4:**
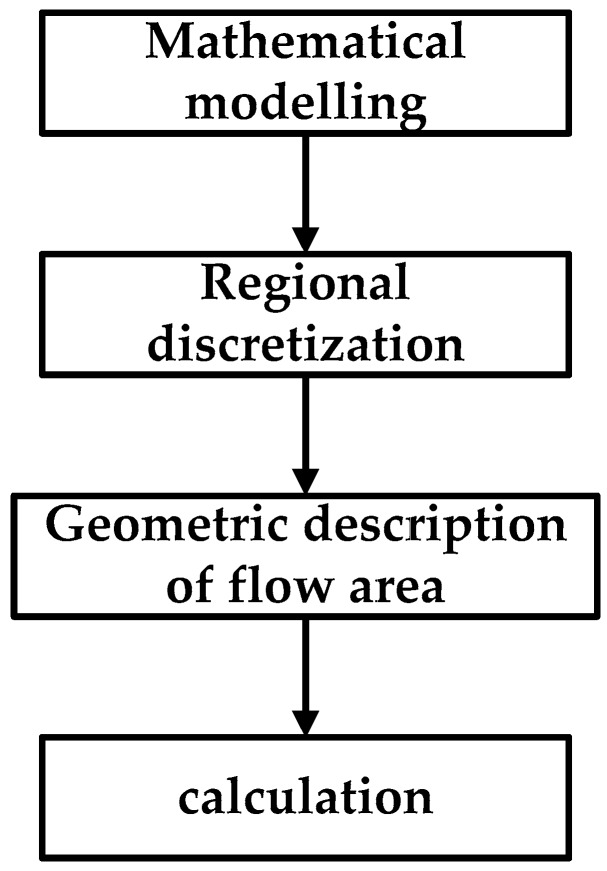
Computational fluid dynamics (CFD) mesh generation and numerical solution process.

CFD is a branch of fluid mechanics. It uses computers as tools and applies various discrete mathematical methods to conduct numerical experiments, computer simulations, and analytical studies on various fluid-mechanics problems. The advantage of CFD lies in its ability to simulate the experimental process from basic physics theorems instead of expensive fluid-dynamics experiment equipment. According to the specific process of CFD numerical simulation analysis [25], we obtained 3D-sized data of the DJI phantom drone (a type of UAV) with a 3D scanning system, converted the 3D information of the UAV into a digital signal that could be processed by the computer, and used CFD to perform UAV mesh generation and numerical solution. The second-order upwind style was selected for calculation to improve accuracy [34,35,36]. The 3D scanning technology was only used to measure the profile data of the UAV fuselage and rotor, and construct a 3D UAV model. This is not a 3D model of the crop-canopy structure. The specific calculation process is shown in Figure 4.

**Figure 5:**
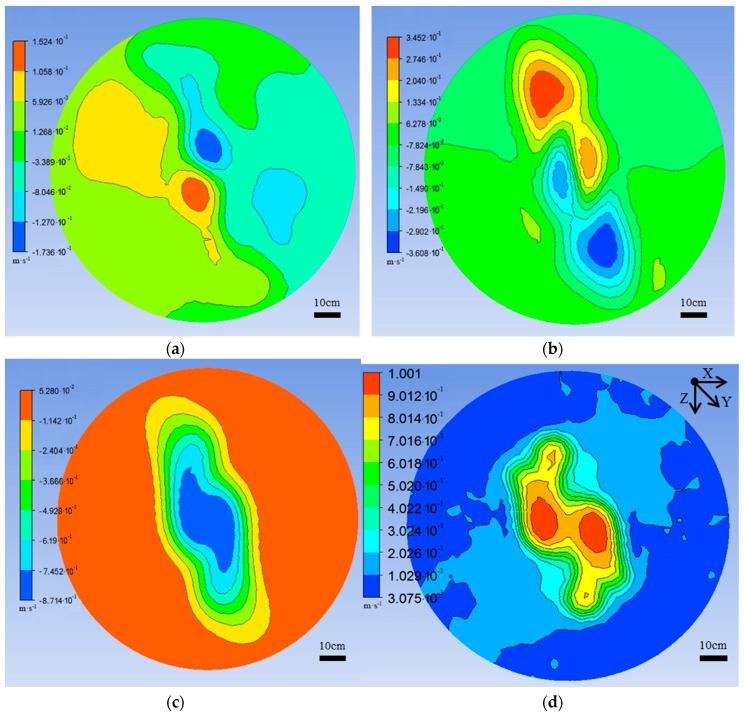
Ground-velocity nephogram distribution results. (**a**) X-axial velocity distribution nephogram; (**b**) Y-axial velocity distribution nephogram; (**c**) Z-axial velocity distribution nephogram; (**d**) combined velocity distribution nephogram.

Physical parameters such as UAV flight area, and rotor-rotation speed and direction, were set by CFD software, and UAV hovering-operation-state simulation analysis was carried out. In the grid simulation area with a diameter of 1.2 m and a height of 1.85 m, the velocity of each grid node is composed of the X-axle, Y-axle, and Z-axle velocity components. The flying height of the UAV was set to 1 m from the ground, and the ground-velocity nephogram distribution result was displayed by CFX’s own post processing module, as shown in Figure 5.

Velocity intensity was analyzed, as shown in Figure 5. When the UAV was hovering at a height of 1 m from the ground, the Z-axial velocity component was much larger than the X-axial and Y-axial velocity components. The combined velocity depends mainly on the axial velocity component. The size of the axial velocity nephogram was elliptical, the central region had the highest wind speed, and it gradually decreased toward the periphery. The results of the combined velocity nephogram distribution show that, due to the opposite rotation of the two pairs of rotors of the quadrotor UAV, forward-rotating rotors generate a downwash flow, so the region with the highest wind speed is distributed directly below the two forward-rotating rotors, wind speed gradually decreases toward the periphery, and is also distributed in an elliptical shape. The long semiaxis of the region is about 0.35 m, and the short semiaxis is about 0.3 m.

#### 2.2.2. Actual Test of Airflow Field

**Figure 6:**
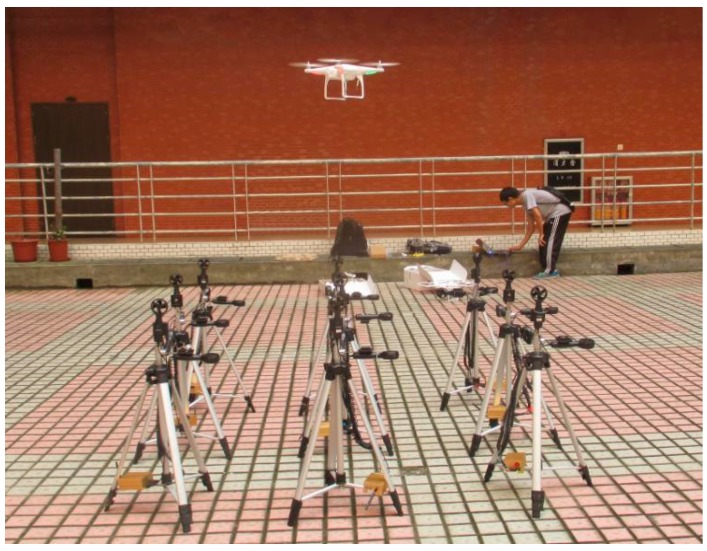
Actual test of downwash flow field.

In order to verify the numerical simulation analysis results of the airflow field, the 3D airflow field testers were used to carry out a real-world test of the downwash airflow field under the hovering operation state of the UAV. Nine 3D airflow field testers were used to test the X-axial, Y-axial, and Z-axial velocity components of the downwash airflow field. The testers were arranged in an array structure of three rows and three columns at equal intervals. The UAV was hovering and flying at a vertical position above the center of the array. Flight height was 1 m from the array plane. When the distance between adjacent testers was 0.6 m, the wind-speed data collected by the testers at the edge of the array were 0 m/s. Therefore, tester spacing was adjusted to 0.5 m, so testers at the edge of the array collected nonzero data, which met the test requirements. In the test, after the flight attitude of the UAV was stabilized, each three-dimensional airflow field tester stopped the test after collecting 100 datasets. The test process is shown in Figure 6.

To reduce the error, the 10 maximum and 10 minimum values were removed from the 100 collected datasets before calculating the average value from the remaining data. The obtained result was solved by interpolation using the four-point spline-interpolation (V4) algorithm. When the adjacent tester spacing was 0.6 m, the edge-tester test result was 0 m/s. Therefore, the interpolation boundary was set to 0.6 m away from the center. The V4 interpolation algorithm is also called the interpolation algorithm based on biharmonic Green’s functions. The difference surface is a linear combination of Green’s functions centered on each sample. The surface is passed through various points by adjusting the weight of each point. Green’s function of the spline satisfies the following biharmonic equation:

(1) $$\frac{d^{4}\phi }{dx^{4}}=6\delta (x)$$

The specific solution of Equation (1) is

(2) $$\phi (x)=|x{|}^{3}$$

When Green’s function is used to interpolate *N* data points, the problem of $w_{i}$ interpolation at $x_{i}$ is

(3) $$\frac{d^{4}w}{d^{4}}=\sum_{j=1}^{N}6\alpha_{j}\delta (x-x_{j})$$

(4) $$w(x_{i})=w_{i}$$

The specific solution to Equations (3) and (4) is that Green’s function is linearly combined around each data point, eliminating the need for a uniform solution.

(5) $$w(x)=\sum_{j=1}^{N}\alpha_{j}{\left|x-x_{j}\right|}^{3}$$

Green’s function of the plane space is shown in Table 1:

Weight value $\alpha_{j}$ is obtained by using the *x* values and w(x) of N points, and the interpolation results are obtained by substituting the weight value into Equation (5).

**Figure 7:**
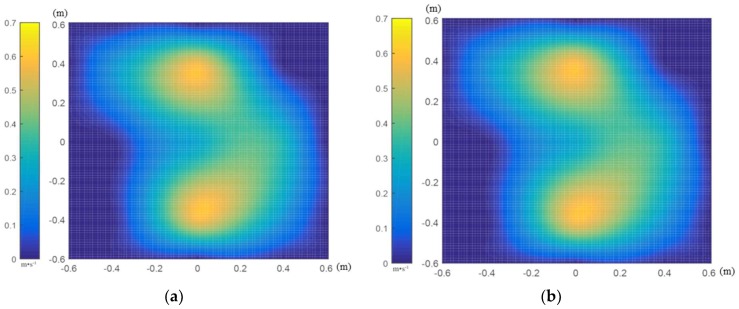

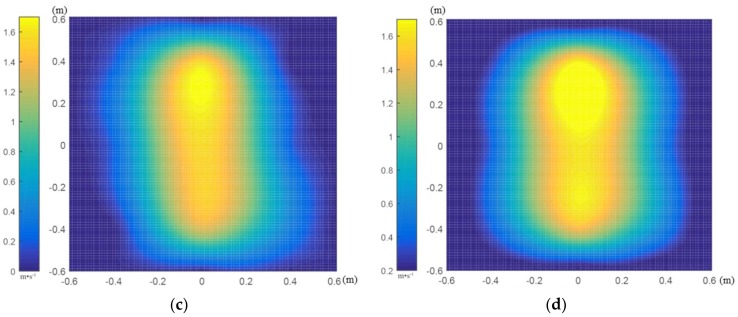
Three-dimensional tester interpolation results when the DJI phantom drone (unmanned aerial vehicle, UAV) hover-flight height is 1 m from 3D airflow field testers. (**a**) X-axial velocity profile; (**b**) Y-axial velocity profile; (**c**) Z-axial velocity profile; (**d**) combined velocity profile.

The data of each 3D airflow field tester were sorted, invalid data were eliminated, valid data were retained, and data results were analyzed and processed. According to the V4 interpolation principle and calculated by MATLAB software, the interpolation results of each 3D airflow field tester node data are displayed in Figure 7.

From the results of Figure 7, we can see that, when the UAV hovering flight height was 1 m from the test plane of the 3D airflow field testers, the Z-axial velocity component of the tester was much larger than the X-axial and Y-axial velocity components. The combined velocity mainly depends on the axial velocity component. In the distribution range, the axial velocity component is elliptical, and the center velocity is the largest and gradually decreases toward the periphery. The combined-velocity distribution results show that the influence range of the UAV downwash airflow field was also elliptical, the center-point velocity was the largest, and peripheral speed was gradually reduced. The long semiaxis of the affected area was about 0.45 m, and the short semiaxis was about 0.4 m. Test results are consistent with the CFD numerical simulation results.

#### 2.2.3. Dual-Band Crop-Growth-Sensor Deployment Method

According to CFD numerical simulation analysis and the actual test results of the 3D airflow field, combined with the test field of view of the dual-band crop-growth sensor, when the dual-band crop-growth sensor was deployed 60 cm away from the UAV rotors, the test area of the crop canopy retained Lambert characteristics, and measurement results were not affected by the airflow field, enabling normal testing. Therefore, we here designed a carbon-fiber sensor support with a length of 60 cm. One end of the support was fixed under the UAV spiral wing, and the other end extends outward along the UAV arm. The sensor was fixed to the rear end of the support through a mechanical structure. The support is connected to the UAV by a cantilever beam structure. We also designed supports of other lengths for experimental comparison.

**Figure 8:**
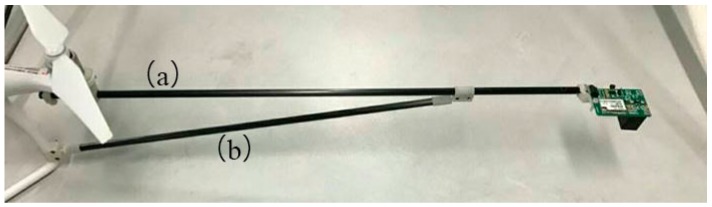
Design of UAV support and damping rod. (**a**) Support; (**b**) damping rod.

In order to avoid the vibration impact of rotor high-speed rotation on the dual-band crop-growth sensor and the support during the flight, a damping rod was designed for shock absorption in order to maintain the stable state of the support and the dual-band crop-growth sensor. The support and damping rod were fixed with a triangular structure to improve the overall stability and shock resistance, as shown in Figure 8. The angle between support and damping rod is very important for the stability of the structure and the balance performance of the aircraft. Therefore, the optimal value of the angle was calculated by static equation analysis.

**Figure 9:**
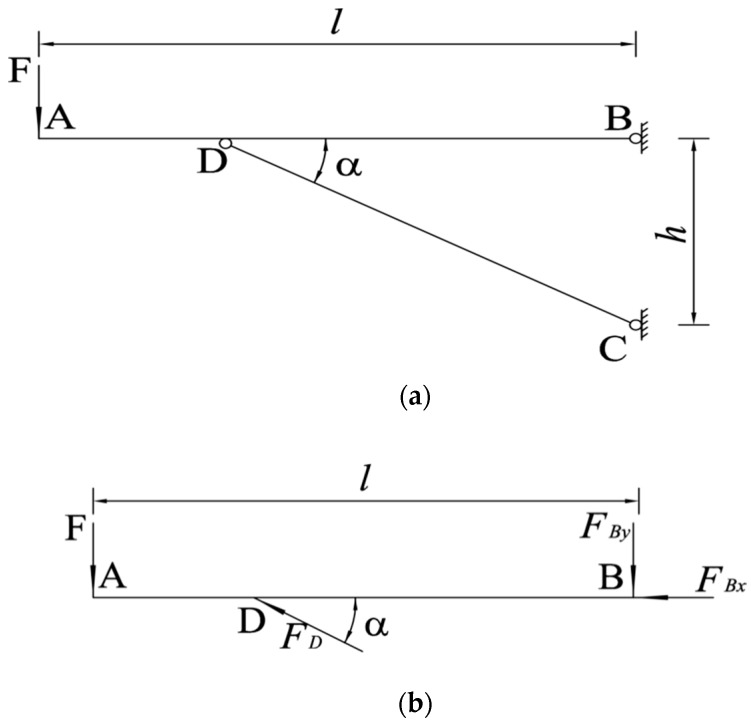
Analysis of the force sustained by UAV sensor support. (**a**) Schematic diagram of the structure of the support and damping rod; (**b**) analysis of the force sustained by each part of the support.

Taking the sensor support as the research object, the analysis diagram of the force sustained by the support is shown in Figure 9.

In Figure 9, AB is the sensor support, CD is the support damping rod, points C and D are the fixed points of the sensor support and the damping rod at the end of the UAV, and the force sustained by the sensor support is analyzed by the following static equations:

(6) $$F=-F_{By}+F_{D}\sin\alpha$$

(7) $$F_{D}\cos\alpha =-F_{Bx}$$

(8) $$Fl=F_{D}\sin\alpha \cdot \frac{h}{\tan\alpha }$$

Simultaneous calculations were performed on Equations (6)–(8) to obtain the following:

(9) $$F_{D}=\frac{Fl}{h\cos\alpha }$$

(10) $$F_{Bx}=-\frac{Fl}{h}$$

(11) $$F_{By}=F\left(\frac{l}{h}\tan\alpha -1\right)$$

In Equations (6)–(11), *F_D_* is the internal force of CD, *F_Bx_* and *F_By_* are the reaction forces in the horizontal and vertical directions of fixed-point B, respectively, *α* is the angle between AB and CD, *h* is the height difference between fixed points B and C, and *l* is the length of AB.

When the length of the UAV support is 60 cm, the value of *α* ranges from 9.5° to 90°. Since a certain length of the support needs to be used for fixing the UAV and dual-band crop-growth sensor, the actual value range of *α* is 13°–90°. From the derivation results of Equations (9)–(11), it can be seen that *F_D_* and *F_By_* decrease with the decrease of *α* , and the smaller the values of *F_D_* and *F_By_* are, the more stable the force sustained by the support structure is. Therefore, the optimal angle between sensor support and damping rod is 13°.

### 2.3. Field Trial

#### 2.3.1. Test Design

Field Trial 1 was conducted at the Baipu Town (32°14′58.88 N 120°45′44.26 E) test base in Rugao City, Jiangsu Province, China, from February to May 2016. The test varieties were Ningmai 13 (V1) and Huaimai 33 (V2), three different gradients of nitrogen fertilizer treatment were set up, which were N_0_ (0 kg/hm^2^), N_1_ (180 kg/hm^2^), and N_2_ (270 kg/hm^2^), and each variety was repeated three times. Each planting area was 30 m^2^ (5 × 6 m). In addition, the application rate of phosphate fertilizer was 120 kg/hm^2^, and the application rate of potassium fertilizer was 135 kg/hm^2^, which was applied once in the base fertilizer. Other cultivation-management measures were the same as those in general high-yield fields.

Field Trial 2 was conducted at the Lingqiao Township (33°35′53.27 N 118°51′11.01 E) Test Base in Huai’an City, Jiangsu Province, China, from July to October 2016. The test varieties were Nanjing 9108 (V1) and Lianjing 10 (V2), four different gradients of nitrogen fertilizer were applied, which were N_0_ (0 kg/hm^2^), N_1_ (120 kg/hm^2^), N_2_ (240 kg/hm^2^), and N_3_ (360 kg/hm^2^), each variety was repeated three times, and each planting area was 30 m^2^ (5 × 6 m). In addition, the application rate of phosphate fertilizer was 105 kg/hm^2^ and was applied once in the base fertilizer, the potassium fertilizer was 135 kg/hm^2^, the base fertilizer was applied 50%, and, at the early boot stage, application was 50%. The other cultivation-management measures were the same as those in general high-yield fields.

#### 2.3.2. Test Method

**Figure 10:**
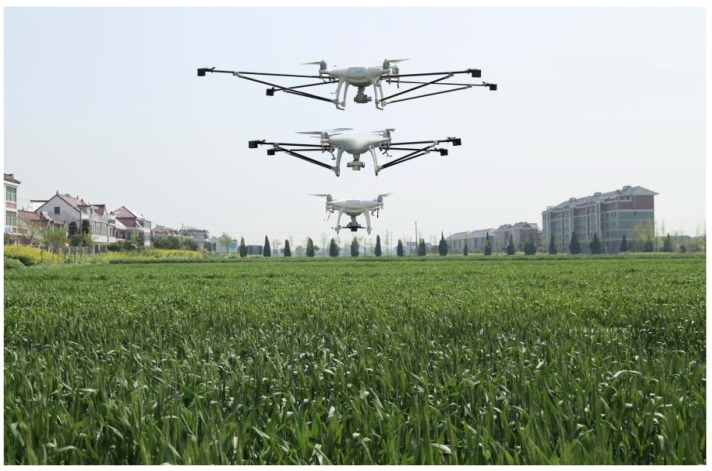
Comparison of field flights with the conditions of three support lengths.

Field Trial 1 was used to test whether the proposed monitoring method can effectively avoid the disturbance range of the UAV downwash airflow field when acquiring data from the crop canopy. Additionally, Field Trial 1 was used to verify the accuracy and stability of the dual-band crop-growth sensor test results. The experiment was carried out in the middle of the wheat jointing stage. The test was carried out on a clear, windless, and cloudless day. Test time was between 10:00 and 14:00. The UAV was flown 1 m above the wheat canopy, and, as shown in Figure 10, the dual-band crop-growth sensor was deployed in three different horizontal distances from the UAV rotors: 0 (i.e., directly below the UAV), 30, and 60 cm. The sensors determined the RVI value of the wheat canopy by measuring three random points in each subarea and repeating the measurement of each point three times to obtain an average value. The ASD FieldSpec HandHeld 2 was used to measure the RVI value of the wheat canopy at the same time.

Field Trial 2 was used to evaluate the applicability and accuracy of UAV-borne spectral sensors for crop-growth parameters. The test was carried out in the tillering, jointing, booting, and heading stages of the rice, test weather was sunny and windless, and test time was between 10:00 and 14:00. In the test, the UAV was made to hover at a height of 1 m above the rice canopy in different test plots, and the dual-band crop-growth sensor was deployed at a horizontal distance of 60 cm from the UAV rotors to obtain the RVI value at the 730 and 815 nm bands of the rice canopy. Three points were randomly measured in each subarea, and the measurement of each point was repeated three times to obtain an average value. The FieldSpec HandHeld 2 and LAI2200 testers were synchronously used to obtain the RVI and LAI values of the rice leaf layer. At the same time, in parallel with the test, the rice sample was destructively sampled, and the sample was placed at 105 °C for 30 min for fixing, then baked at 80 °C to constant weight, and weighed to obtain the LDW. After the sample was pulverized, the LNA was determined by the Kjeldahl method.

#### 2.3.3. Analysis of Field-Test Data

The field-test datasets were statistically analyzed with Microsoft Excel 2010 software; the correlation of the model was evaluated by the coefficient of determination (R^2^) and root mean square error (RMSE).

## 3. Results and Discussion

### 3.1. Field-Test Results

**Figure 11:**
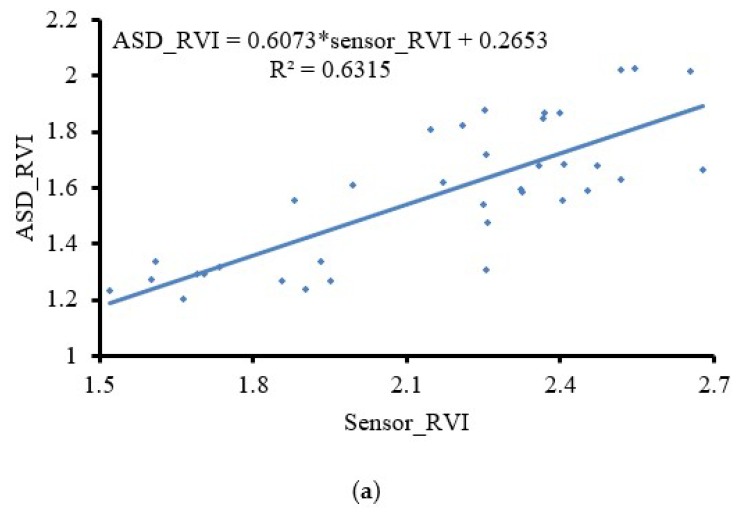

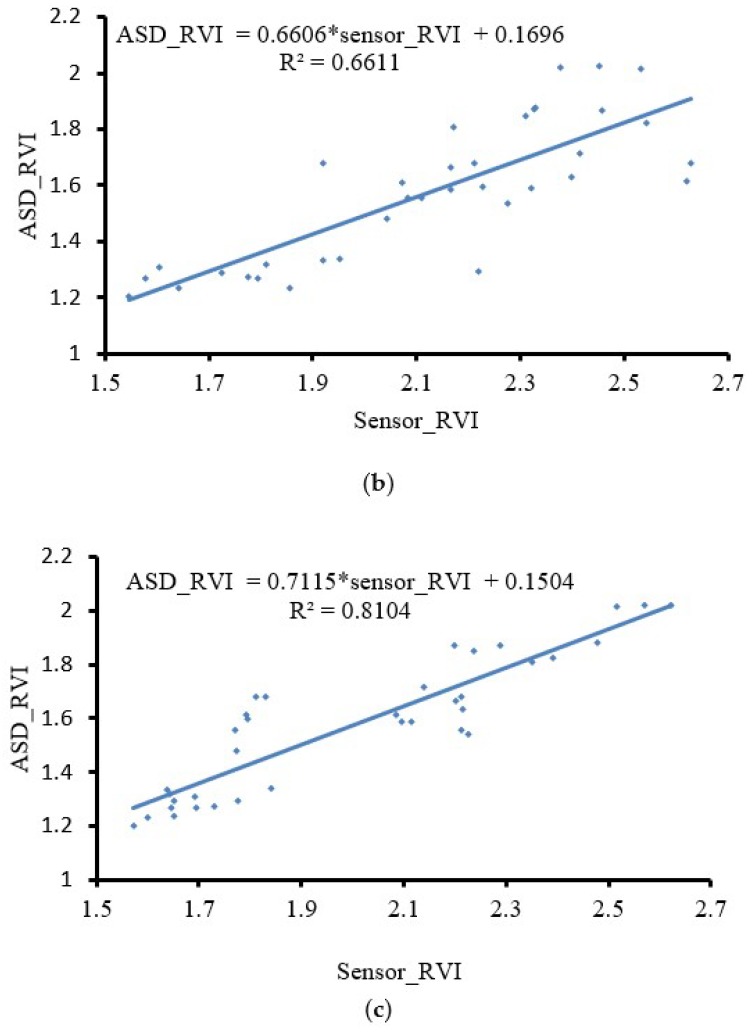
Fitting curves for the sensor and ASD data. (**a**) Sensor located under the UAV; (**b**) sensor located 30 cm from the rotor; (**c**) sensor located 60 cm from the rotor.

Figure 11 shows the test results of Field Trial 1, in which the dual-band crop-growth sensor was located at different positions relative to the UAV in the middle of the wheat-jointing stage. Parts a, b, and c of Figure 11 show the simple linear fitting results of the RVI test value of the UAV-borne spectral sensor when the length of the support was 0, 30, and 60 cm, respectively, and the RVI value of the handheld ASD FieldSpecHandHeld 2 spectrometer in the corresponding growth period. When the length of the support was 0 and 30 cm, the results were close, and R^2^ values were 0.63 and 0.66, respectively. When the length of the support was extended to 60 cm, the curve fitting degree was obviously improved; R^2^ was 0.81, and RMSE was 0.38.

It can be seen from the above test results that the farther the dual-band crop growth sensor was deployed from the UAV rotors, the better the correlation between the test data and the ASD test results. According to CFD numerical simulation results and 3D airflow field-test results, when the dual-band crop-growth sensor was deployed directly below the UAV, the sensor-test field of view was completely within the disturbance range of UAV rotor downwash airflow field. When the dual-band crop-growth sensor was deployed 30 cm away from the UAV rotors, the sensor-test field of view included both the disturbance and nondisturbance zone of the rotor downwash airflow field. The correlation of the data results improved slightly, but it was still not ideal. When the dual-band crop-growth sensor was deployed 60 cm away from the rotors of the UAV, the sensor-test field was completely in the nondisturbance zone, and the correlation of the data results was significantly improved. In summary, the downwash airflow field generated by the rotation of the UAV rotors has a certain influence on the results of the dual-band crop-growth sensor. The proposed UAV-borne spectral sensor crop-growth monitoring method can effectively target areas of the crop canopy outside the disturbance range of the UAV downwash airflow field.

**Figure 12:**
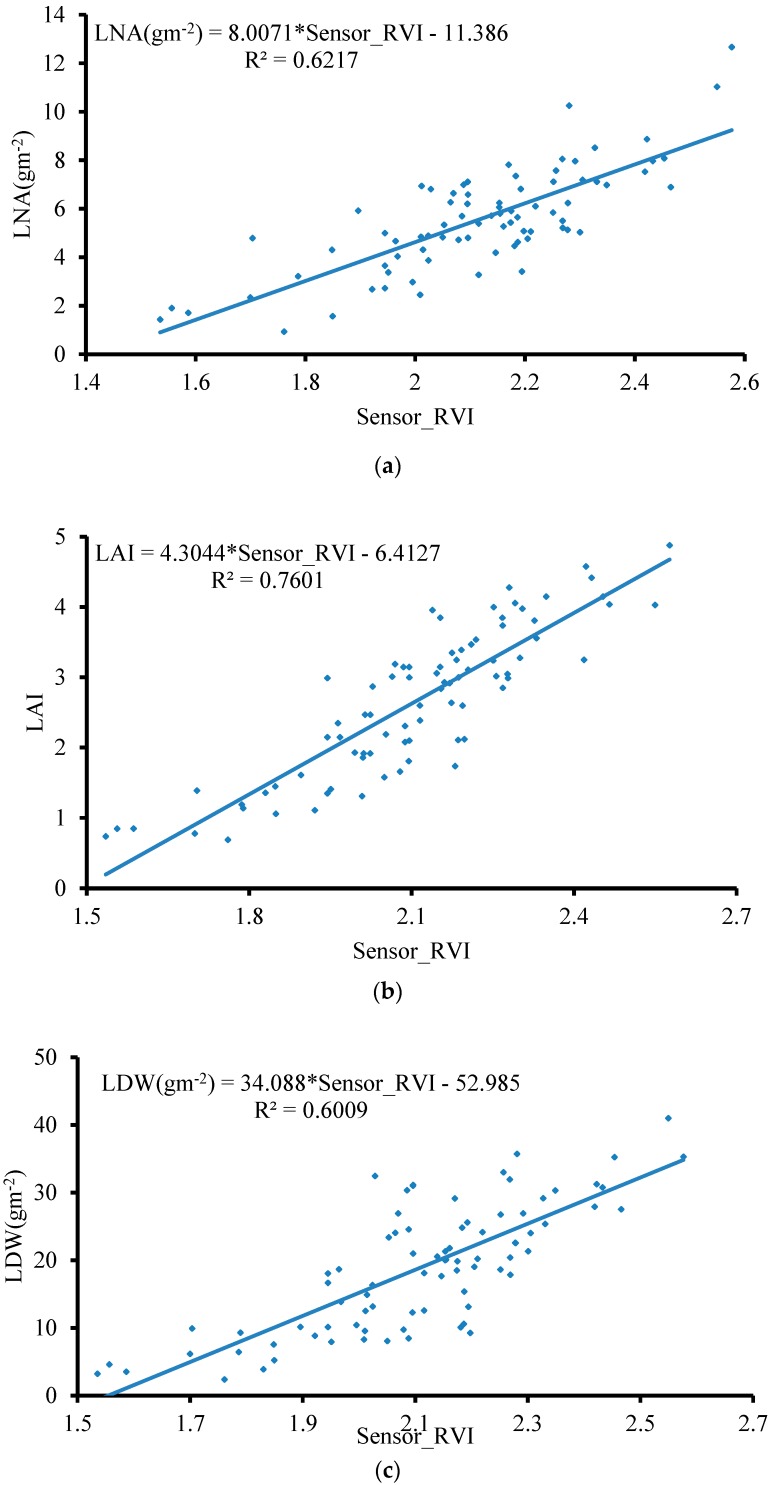
Spectral model for the UAV-borne crop-growth monitoring system. (**a**) LNA–RVI fitting curve; (**b**) LAI–RVI fitting curve; (**c**) LDW–RVI fitting curve.

In Field Trial 2, the flying height of the UAV was 1 m from the rice canopy, the dual-band crop-growth sensor was deployed 60 cm away from the UAV rotors, and measurements were taken throughout the entire growth period of rice. Figure 12 shows the linear fitting results of the RVI values obtained by our method, and the LNA, LAI, and LDW obtained from the field test and the indoor chemical analysis test. R^2^ values were 0.62, 0.76, and 0.60, and RMSE values were 2.28, 1.03, and 10.73, respectively. Using the proposed UAV-borne spectral sensor crop-growth monitoring method, rice-growth parameters in the entire growth period could accurately be obtained.

### 3.2. Discussion

UAVs have the characteristics of simple operation and high efficiency. At present, there is little targeted research on monitoring crop growth by UAV-borne dual-band crop-growth sensors. Under a low-altitude hovering operation, the downwash airflow field generates strong disturbance on the crop canopy that disrupts the Lambertian reflection characteristics of the crop canopy and thus has a serious impact on the accuracy of the test, even causing the test to fail. Therefore, we used CFD numerical simulation and real-world 3D airflow field testing to analyze the spatial distribution of the downwash airflow field when the UAV is hovering at a height of 1 m above the crop canopy and determine the disturbance range on the crop canopy. Most of the current studies simply mounted the energy-type spectrum sensor on UAVs, lacking consideration for the disturbance influence of the crop canopy by the UAV downwash airflow field during the test [37]. Our study filled the gap in these studies. Our test showed that the influence range of the downwash airflow field of the UAV is elliptical, wind speed at the center point is the largest, and gradually decreases toward the periphery. The long semiaxis was about 0.45 m, and the short semiaxis was about 0.4 m. According to the distribution range of the downwash airflow field, we designed a support with a length of 0.6 m to deploy a dual-band crop-growth sensor so that the test field of view is extended beyond the distribution range of the downwash airflow field, and the disturbance effect of the downwash airflow field on the crop canopy was avoided. When the flight height of the UAV was kept at 1 m from the wheat canopy and the dual-band crop-growth sensor was deployed 0.6 m from the rotors, the linear fit R^2^ of the test output RVI value and the handheld ASD Fieldspec2 spectrometer, the test RVI value was 0.81, and RMSE was 0.38. Therefore, the UAV-borne spectral sensor crop-growth monitoring method can effectively target areas of the crop canopy outside the disturbance range of the UAV downwash airflow field.

In the test process of the 3D airflow field testers, the wind-speed results of different dimensions measured by the testers were larger than the CFD numerical-simulation results. The main reason was that the test plane of the 3D airflow field testers was 60 cm away from the ground, and the arrangement was lattice. When the downwash airflow diffused downward through the lattice plane, the direction of the airflow changed. In the CFD numerical simulation analysis, the downwash airflow directly reached the ground plane, so the distribution states of the real-world test and numerical simulation differed. Although the analyzed UAV in this paper is a DJI Phantom drone, the proposed UAV-borne spectral sensor crop-growth monitoring method can be applied to various types of multirotor UAV structures.

## 4. Conclusions

1. We identified the UAV-borne spectral sensor crop-growth monitoring method, used CFD numerical simulation and an actual test of 3D airflow field to determine the distribution range of the UAV downwash airflow field above the surface of the crop canopy, and designed sensor supports to target areas of the crop canopy outside the disturbance range of the UAV downwash airflow field.

2. When the flying height of the UAV was 1 m from the crop canopy, the influence range of the downwash airflow field of the UAV was elliptical, central wind speed was the largest and gradually decreased toward the periphery, the long semiaxis was about 0.45 m, and the short semiaxis was about 0.4 m. When the designed sensor support length was 60 cm, the sensor-test field of view was completely outside the disturbance range of the UAV downwash airflow field.

3. The wheat test showed that, when the dual-band crop-growth sensor was deployed at 0, 30, and 60 cm from the UAV, the linear fit R^2^ of the RVI value obtained by our method, and the RVI value measured by the ASD FieldSpec HandHeld 2 spectrometer, was 0.63, 0.66, and 0.81, respectively. When the length of the support was 60 cm, the fitting degree was obviously improved. The UAV-borne spectroscopy sensor crop-growth monitoring method can effectively avoid the disturbance range of the UAV downwash airflow field on the crop canopy.

4. The rice experiment showed that the RVI value measured by the UAV-borne spectral sensor had a good linear fitting relationship with the LNA, LAI, and LDW obtained from the field test and the indoor chemical analysis test. R^2^ values were 0.62, 0.76, and 0.60, respectively, and RMSE values were 2.28, 1.03, and 10.73, respectively. Using the UAV-borne spectral sensor crop-growth monitoring method, rice-growth parameters during the entire growth period could be accurately obtained.

## Figures and Tables

**Table 1:** Function in flat space.

Dimension m	Green’s Function $\phi_{m}(x)$
1	${\left|x\right|}^{3}$
2	${\left|x\right|}^{2}(\ln|x|-1)$
3	|x|

## References

[1] Pan J., Liu Y., Zhong X., Lampayan R.M., Singleton G.R., Huang N., Liang K., Peng B., Tian K. (2017). Grain yield, water productivity and nitrogen use efficiency of rice under different water management and fertilizer-N inputs in South China. Agric. Water Manag..

[2] Cambouris A.N., Zebarth B.J., Ziadi N., Perron I. (2014). Precision Agriculture in Potato Production. Potato Res..

[3] Guo J.H., Wang X., Meng Z.J., Zhao C.J., Zhen-Rong Y.U., Chen L.P. (2008). Study on diagnosing nitrogen nutrition status of corn using Greenseeker and SPAD meter. Plant Nutr. Fertil. Sci..

[4] Grohs D.S., Bredemeier C., Mundstock C.M., Poletto N. (2009). Model for yield potential estimation in wheat and barley using the GreenSeeker sensor. Eng. Agrícola.

[5] Ali A.M., Thind H.S., Sharma S., Varinderpal-Singh V. (2014). Prediction of dry direct-seeded rice yields using chlorophyll meter, leaf color chart and GreenSeeker optical sensor in northwestern India. Field Crop. Res..

[6] Moya I., Camenen L., Evain S., Goulas Y., Cerovic Z.G., Latouche G., Flexas J. (2004). A new instrument for passive remote sensing: 1. Measurements of sunlight-induced chlorophyll fluorescence. Remote Sens. Environ..

[7] Youngryel R., Dennisd B., Joseph V., Ma S., Matthias F., Ilse R.M., Ted H. (2011). Testing the performance of a novel spectral reflectance sensor, built with light emitting diodes (LEDs), to monitor ecosystem metabolism, structure and function. Agric. For. Meteorol..

[8] Martin D.E., Lan Y.B. (2012). Laboratory evaluation of the GreenSeekerTM handheld optical sensor to variations in orientation and height above canopy. Int. J. Agric. Biol. Eng..

[9] Ali A.M., Abouamer I., Ibrahim S.M. (2018). Using GreenSeeker Active Optical Sensor for Optimizing Maize Nitrogen Fertilization in Calcareous Soils of Egypt. Arch. Agron. Soil Sci..

[10] Vohland M., Besold J., Hill J., Fründ H.C. (2011). Comparing different multivariate calibration methods for the determination of soil organic carbon pools with visible to near infrared spectroscopy. Geoderma.

[11] Kusumo B.H., Hedley M.J., Hedley C.B., Hueni A., Arnold G.C. (2010). The use of Vis-NIR spectral reflectance for determining root density: Evaluation of ryegrass roots in a glasshouse trial. Eur. J. Soil Sci..

[12] Nawar S., Buddenbaum H., Hill J., Kozak J., Mouazen A.M. (2016). Estimating the soil clay content and organic matter by means of different calibration methods of vis-NIR diffuse reflectance spectroscopy. Soil Tillage Res..

[13] Cao Q., Miao Y., Wang H., Huang S., Cheng S., Khosla R. (2013). Non-destructive estimation of rice plant nitrogen status with Crop Circle multispectral active canopy sensor. Field Crop. Res..

[14] Bonfil D.J. (2016). Wheat phenomics in the field by RapidScan: NDVI vs. NDRE. Isr. J. Plant Sci..

[15] Lu J., Miao Y., Wei S., Li J., Yuan F. (2017). Evaluating different approaches to non-destructive nitrogen status diagnosis of rice using portable RapidSCAN active canopy sensor. Sci. Rep..

[16] Miller J.J., Schepers J.S., Shapiro C.A., Arneson N.J., Eskridge K.M., Oliveira M.C., Giesler L.J. (2018). Characterizing soybean vigor and productivity using multiple crop canopy sensor readings. Field Crop. Res..

[17] Zhou Z., Andersen M.N., Plauborg F. (2016). Radiation interception and radiation use efficiency of potato affected by different N fertigation and irrigation regimes. Eur. J. Agron..

[18] Krienke B., Ferguson R.B., Schlemmer M., Holland K., Marx D., Eskridge K. (2017). Using an unmanned aerial vehicle to evaluate nitrogen variability and height effect with an active crop canopy sensor. Precis. Agric..

[19] Shafian S., Rajan N., Schnell R., Bagavathiannan M., Valasek J., Shi Y. (2018). Unmanned aerial systems-based remote sensing for monitoring sorghum growth and development. PLoS ONE.

[20] Schirrmann M., Giebel A., Gleiniger F., Pflanz M., Lentschke J. (2016). Monitoring Agronomic Parameters of Winter Wheat Crops with Low-Cost UAV Imagery. Remote Sens..

[21] Zheng H., Zhou X., Cheng T., Yao X., Tian Y., Cao W., Zhu Y. Evaluation of a UAV-based hyperspectral frame camera for monitoring the leaf nitrogen concentration in rice. Proceedings of the Geoscience and Remote Sensing Symposium.

[22] Stroppiana D., Villa P., Sona G., Ronchetti G., Candiani G., Pepe M., Busetto L., Migliazzi M., Boschetti M. (2018). Early season weed mapping in rice crops using multi-spectral UAV data. Int. J. Remote Sens..

[23] Wu M., Huang W., Niu Z., Wang Y., Wang C., Li W., Hao P., Yu B. (2017). Fine crop mapping by combining high spectral and high spatial resolution remote sensing data in complex heterogeneous areas. Comput. Electron. Agric..

[24] Sandwell D.T. (2013). Biharmonic spline interpolation of GEOS-3 and SEASAT altimeter data. Geophys. Res. Lett..

[25] Ni J., Yao L., Zhang J., Cao W., Zhu Y., Tai X. (2017). Development of an Unmanned Aerial Vehicle-Borne Crop-Growth Monitoring System. Sensors.

[26] Feng W., Zhu Y., Tian Y., Cao W., Yao X., Li Y. (2008). Monitoring leaf nitrogen accumulation in wheat with hyper-spectral remote sensing. Eur. J. Agron..

[27] Yan Z., Dongqin Z., Xia Y., Yongchao T., Weixing C. (2007). Quantitative relationship between leaf nitrogen accumulation and canopy reflectance spectra in wheat. Aust. J. Agric. Res..

[28] Yan Z., Yingxue L., Wei F., Yongchao T., Xia Y., Weixing C. (2006). Monitoring leaf nitrogen in rice using canopy reflectance spectra. Can. J. Plant Sci..

[29] Szuvandzsiev P., Helyes L., Lugasi A., Szántó C., Baranowski P., Pék Z. (2014). Estimation of antioxidant components of tomato using VIS-NIR reflectance data by handheld portable spectrometer. Int. Agrophys..

[30] Battay A.E., Mahmoudi H. (2016). Linear spectral unmixing to monitor crop growth in typical organic and inorganic amended arid soil. IOP Conf. Ser. Earth Environ. Sci..

[31] Fang H., Li W., Wei S., Jiang C.J.A., Meteorology F. (2014). Seasonal variation of leaf area index (LAI) over paddy rice fields in NE China: Intercomparison of destructive sampling, LAI-2200, digital hemispherical photography (DHP), and AccuPAR methods. Agric. For. Meteorol..

[32] Sandmann M., Graefe J., Feller C. (2013). Optical methods for the non-destructive estimation of leaf area index in kohlrabi and lettuce. Sci. Hortic..

[33] Kobayashi H., Ryu Y., Baldocchi D.D., Welles J.M., Norman J.M. (2013). On the correct estimation of gap fraction: How to remove scattered radiation in gap fraction measurements?. Agric. For. Meteorol..

[34] Jiradilok V., Gidaspow D., Damronglerd S., Koves W.J., Mostofi R. (2006). Kinetic theory based CFD simulation of turbulent fluidization of FCC particles in a riser. Chem. Eng. Sci..

[35] Blocken B., Stathopoulos T., Carmeliet J. (2007). CFD simulation of the atmospheric boundary layer: Wall function problems. Atmos. Environ..

[36] Nakata T., Liu H., Bomphrey R.J. (2015). A CFD-informed quasi-steady model of flapping wing aerodynamics. J. Fluid Mech..

[37] Li S., Ding X., Kuang Q., Ata-UI-Karim S.T., Cheng T., Liu X., Tian Y., Yan Z., Cao W., Cao Q. (2018). Potential of UAV-Based active sensing for monitoring rice leaf nitrogen satus. Front. Plant Sci..

